# Effect of endometrial injury during menstruation on clinical outcomes in frozen–thawed embryo transfer cycles: A randomized control trial

**DOI:** 10.1111/jog.14193

**Published:** 2020-01-30

**Authors:** Zhixia Tang, Mingyun Hong, Fang He, Dayan Huang, Zhijun Dai, Henghua Xuan, Hong Zhang, Weipei Zhu

**Affiliations:** ^1^ Department of Obstetrics and Gynecology The Second Affiliated Hospital of Soochow University Suzhou China; ^2^ Reproductive Medicine Center, Maternity and Child Health Hospital of Anhui Province, The Maternal and Child Health Clinical College Anhui Medical University Hefei China

**Keywords:** endometrial injury, frozen–thawed embryo transfer, implantation rate, pregnancy rate

## Abstract

**Aim:**

To explore the clinical effect of endometrial injury (EI) on the third day of the menstrual cycle before frozen–thawed embryo transfer (frozen–thawed ET) on patients experienced two or more implantation failures.

**Methods:**

A total of 200 patients who suffered at least two failed hormone‐replacement therapies and frozen–thawed ET were randomly divided into two groups: EI group and control group (*n* = 100 in each group). Patients in the EI group received local EI with a Pipelle catheter on the third day of the menstrual cycle before frozen–thawed ET. Primary outcomes were live birth, clinical pregnancy and implantation rates. Secondary outcomes were biochemical, multiple and ectopic pregnancy rates and abortion rates.

**Results:**

The rate of live birth in EI group (51.00%) was significantly higher than that of control group (36.00%) (*P* = 0.032). Clinical pregnancy and implantation rates in EI group were significantly higher comparing to control group (64.00% vs 48.00%, *P* = 0.023 and 46.74% vs 30.11%, *P* = 0.001). The rate of multiple pregnancy in EI group (37.50%) was significantly higher than that of control group (18.75%) (*P* = 0.031). No significant difference in ectopic pregnancy rate and abortion rate was observed between EI group and control group.

**Conclusion:**

Applying EI to patients experienced two or more implantation failures on the third day of the menstrual cycle before frozen–thawed ET can improve clinical outcomes.

## Introduction

Successful implantation depends on both embryo quality and endometrial environment.^1^ It is commonly seen an implantation failed after receiving embryos with good quality, indicating the pivotal role of endometrial receptivity in the implantation process.

There are increasing studies focus on performing endometrial injury (EI) in luteal phase to improve endometrial receptivity. However, there is no consensus in the field up to date and the benefit of EI prior to embryo transfer is controversial. Specifically, some studies demonstrated that performing EI in luteal phase prior to in vitro fertilization (IVF)/ICSI improved implantation and clinical pregnancy rates,^2^, ^3^, ^4^, ^5^, ^6^, ^7^, ^8^, ^9^, ^10^ in the meanwhile, there are some studies denied such benefit of EI.^11^, ^12^, ^13^, ^14^, ^15^, ^16^ It is important to point out that most previous studies focused on fresh embryo transfer.

Frozen–thawed embryo transfer (frozen–thawed ET) is a procedure that involves endometrial preparation and the transfer of thawed embryos obtained through a previous IVF cycle. It mainly applies to patients with supernumerary embryos and canceled fresh embryo transfer due to the risks such as ovarian hyperstimulation syndrome and thin endometrium.^17^

The effect of EI before using frozen–thawed ET cycles has not been well explored. With limited proceeding studies, it seems like EI did not have any beneficial effects on an unselected group of women undergoing frozen–thawed ET.^18^, ^19^ However, Kanazawa *et al*. suggested that EI had a positive effect on pregnancy rate prior to frozen–thawed ET cycles in patients suffered repeated implantation failures (RIF).^20^

Current studies in the field mainly focused on performing EI in luteal phase prior to frozen–thawed ET. This conventional procedure usually takes two menstrual cycles which brings more individual differences and other uncertainties to the situation. Comparing to luteal phase, we hypothesized that the early stage of menstrual cycle might be a better time point to perform EI. This new procedure only requires one menstrual cycle and a more consistent and better‐controlled injury due to a thinner one endometrium.

Therefore, we proposed a prospective randomized controlled trial to evaluate the effectiveness of EI in infertility women undergoing frozen–thawed ET cycle. The purpose of our study is to explore the clinical effect of performing EI on the third day of the menstrual cycle before undergoing frozen–thawed ET of patients suffered two or more implantation failures.

## Methods

### Study design and participants

This prospective randomized controlled trial conducted at Reproductive Center of Anhui Province Maternity and Child Health Hospital between October 2017 and February 2018. All participants were followed‐up to 1 year. This study was approved by the Institutional Ethics Committee Review Board of Anhui Medical University affiliated Maternity and Child Health Hospital. The registration number on http://www.clinicaltrials.gov was ChiCTR‐IPR‐17014013. All participants recruited to the study were fully counseled and consented.

The inclusion criteria were: patients indicated for frozen–thawed ET, with serum progesterone level < 1.2 ng/mL on the third day of the menstrual cycle, at least two or more previous implantation failures, normal morphology of uterine cavity, The exclusion criteria were: history with pelvic surgery history, history with difficult ET and aged more than 40 years; intrauterine abnormality (severe adhesions, uterine polyp, submucosal fibroma), body mass index (BMI) > 27 kg/m^2^, hydrosalpinx, endometriosis and receiving oral contraception drugs recently.

### Randomization and allocation

The study was explained to all eligible patients who were given a patient information sheet. Randomization was done simply using sealed envelopes before undergoing EI. After randomization, physicians and participants were aware of the trail‐group assignments.

### Endometrial injury

In the EI group, patients received endometrial soft scratch on the third day of the menstrual cycle proceeding frozen–thawed ET cycle. EI was performed following standard approach using a Pipelle catheter (Beijing Saipu Jiuzhou Science and Technology Development Company). The Pipelle catheter was introduced through the cervix up to the uterine cavity, and then rotated 360° and moved up and down four times after withdrawing the piston. Simultaneously, endometrial tissue was stained with hematoxylin and eosin, and examined under microscope in order to evaluate the size and level of the injury introduced by the endometrial sampler and to verify the proliferative state of endometrium. The patients in control groups received no intervention. All procedures were performed by two specifically trained senior doctors.

### Endometrial preparation

All patients from both groups followed our standard endometrial preparation protocol for frozen–thawed ET cycles with hormone‐replacement therapy for approximately 14 days. Specifically, patients started estradiol valerate 6 mg daily from day 3 of the cycle. Transvaginal ultrasound evaluation of the endometrium and serial serum estradiol measurements was monitored. The dose of estradiol valerate would be increased to 8 mg daily if the endometrial thickness was less than 8 mm. When the thickness of endometria was ≥8 mm and the concentration of estradiol exceeded 200 pg/mL, the patients began to receive intramuscular progesterone 60 mg daily and frozen–thawed ET was performed 3 or 5 days later, depending on the embryo's stage of development.

### Endometrial pattern

The patterns of endometrium were classified according to Zhao's grading.^21^ Three different patterns could be distinguished: (i) a triple‐line pattern consisting of a central hyperechoic line surrounded by two hypoechoic layers; (ii) an intermediate isoechogenic pattern with the same reflectivity as the surrounding myometrium and a poorly defined central echogenic line; (iii) homogenous, hyperechogenic endometrium.

### Embryo grade and transfer

The cleavage embryos were classified according to Steer's grading.^22^ Grade 1 or 2 embryos were considered to be good‐quality embryos. The blastocyst were graded before freezing with the Gardner and Schoolcraft system.^23^ For fully developed blastocysts, a second scoring was performed by microscope to assess the inner cell mass (ICM). Specifically, we used the following descriptions: (i) tightly packed with many cells; (ii) loosely grouped with several cells and (iii) very few cells. The degree of blastocyst expansion ≥3 and grade A or B of both ICM were considered of good quality. All transferred blastocysts were performed noninvasive chromosome screening by collecting the spent medium samples on blastocyst and all identified as normal.^24^

All embryo transfers were performed under ultrasound guiding. Patients were asked to fill their bladder to provide an acoustic window for uterus visualization. The catheter tip (Wallace, Smiths Medical) was placed 1.0–1.5 cm below the apex of the uterus cavity. In order to prevent uterine contraction, care should be taken to avoid contact of the transfer catheter with the uterine fundus. Hormonal treatment was continued at least till pregnancy test was performed on 14 days after transfer.

### Outcome measure

The primary outcomes were live birth, clinical pregnancy and implantation rates and secondary outcomes were biochemical and multiple pregnancy, miscarriage and ectopic pregnancy rates. Live birth rate was defined by number of deliveries that resulted in a live born after embryo transfer. Clinical pregnancy rate was defined by ultrasound confirmation of gestational sac approximately 5 weeks after embryo transfer. Implantation rate was achieved by dividing the sacs number observed on transvaginal ultrasound scan by the number of transferred embryos. Biochemical pregnancy rate was defined as positive hCG test 14 days after embryo transfer. Multiple pregnancy rate was defined as the number of multiple pregnancies divided by total number of clinical pregnancies. Miscarriage rate was defined as miscarriages number before 20 weeks divided by the number of clinical pregnancies. Ectopic pregnancy rate was defined as the number of patients with ectopic pregnant divided by the number of all confirmed pregnancies.

### Sample size calculation

A power analysis suggested by Barash *et al*. with 30% difference in clinical pregnancy rate demonstrated that our study required 49 patients per group to reach the significance of 5% and a power of 80% in this prospective randomized control design.^2^

### Patient selection

**Figure 1:**
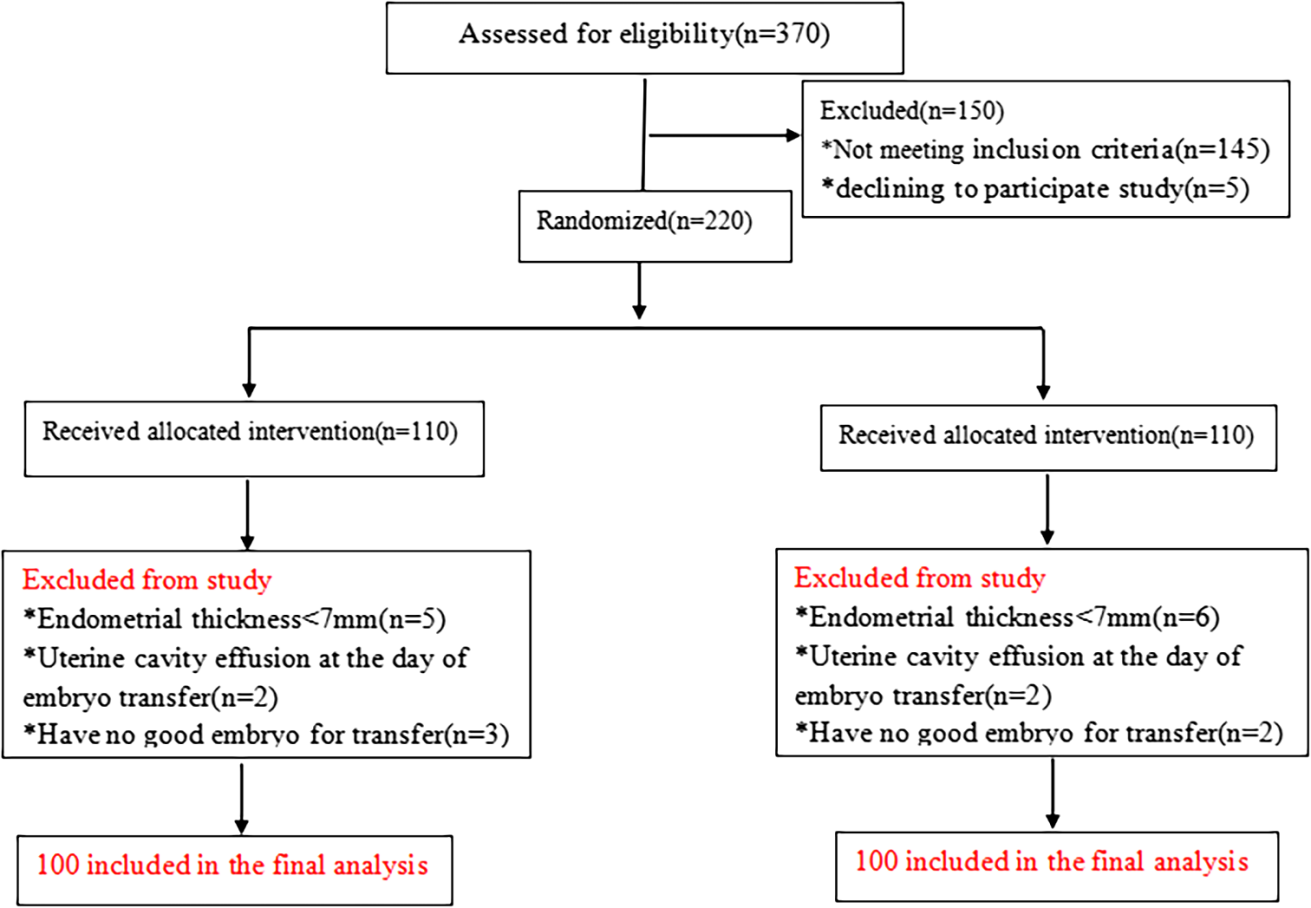
Flow chart of the study recruitment process. The exclusion criteria were: history with pelvic surgery history, history with difficult ET and aged more than 40 years; intrauterine abnormality (severe adhesions, uterine polyp, submucosal fibroma), body mass index (BMI) > 27 kg/m^2^, hydrosalpinx, endometriosis and receiving oral contraception drugs recently.

Initially 370 eligible participants were included in the study for criteria screening. Patients excluded because of not meeting inclusion criteria (*n* = 145) or declining to participate (*n* = 5). The remaining 220 participants were randomly assigned into two groups: EI group (*n* = 110) and control group (*n* = 110). After examination, 10 patients from each group were excluded because of following conditions: endometrial thickness was less than 7 mm, uterine cavity effusion and did not have good quality – according to Veeck classification, embryos for transfer. At the beginning of the study, 100 patients in each group were recruited and analyzed respectively (Fig. 1).

### Statistical analysis

All statistical calculations were performed by spss software (Statistical Package for the Social Sciences Version 21.0). Student^'^s *t*‐test was used for comparing quantitative variables and Chi‐square test was used to compare categorical data. *P* < 0.05 was considered statistically significant.

## Results

In our study, a total of 200 participants who received frozen–thawed ET after two or more implantation failures were divided into two groups: EI group (*n* = 100) and control group (*n* = 100). Baseline characteristics of each group including age, duration of infertility, BMI, basal FSH, endometrial thickness at progesterone initiation day, the pattern A/B of endometrium at progesterone initiation day, number of transferred embryos, ratio of cleavage stage and quality of embryos are shown in Table 1. There was no significant difference in terms of baseline characteristics between two groups. Endometrial injuries were performed successfully in all patients. The potential complications were carefully monitored and no significant vaginal bleeding, fever, abdominal pain and other complications were found in EI group.

**Table 1:** Comparison of baseline characteristics in two groups

	Endometrial injury group (*n* = 100)	Control group (*n* = 100)	*P* value
Age (years) (mean ± SD)	30.98 ± 3.65	30.84 ± 3.95	0.795
Duration of infertility (years) (mean ± SD)	3.55 ± 1.79	3.72 ± 1.86	0.511
BMI (kg/m^2^) (mean ± SD)	22.45 ± 2.28	22.61 ± 2.71	0.642
Basal FSH (mIU/mL) (mean ± SD)	7.24 ± 1.78	7.24 ± 1.77	0.986
Endometrial thickness at progesterone initiation on day (mm) (mean ± SD)	9.45 ± 1.75	9.37 ± 1.46	0.713
Pattern A/B of endometrium at progesterone initiation day (*n*, %)	90 (90.00)	84 (84.00)	0.293
Number of embryo transfer (mean ± SD)	1.84 ± 0.37	1.86 ± 0.59	0.773
Cleavage stage, *n* (%)	57 (57.00)	49 (49.00)	0.321
Blastocyst stage, *n* (%)	43 (43.00)	51 (51.00)	
More than one good quality embryo, *n* (%)	60 (60.00)	59 (59.00)	1.000
Previous embryo transfer cycles (mean ± SD)	2.57 ± 1.15	2.55 ± 1.07	0.539
Twice, *n* (%)	67 (67.00)	72 (72.00)	
More than two times, *n* (%)	33 (33.00)	28 (28.00)	

Our study indicated that performing EI on the third day of the menstrual cycle on patients with two or more times implantation failures before frozen–thawed ET could improve clinical outcomes. Specifically, the rate of live birth in EI group (51.00%) was significantly higher than control group (36.00%) (*P* = 0.032). In addition, clinical pregnancy and implantation rates were significantly increased in EI group compared to control group (64.00% vs 48.00%, *P* = 0.023 and 46.74% vs 30.11%, *P* = 0.001). Furthermore, the multiple pregnancy rate in EI group (37.50%) was also significantly enhanced than that of control group (18.75%) (*P* = 0.031). At last but not the least, the biochemical pregnancy rate in EI group (66.00%) was significantly higher when comparing to control group (49.00%) (*P* = 0.015). In addition, our study suggested that there was no difference in ectopic pregnancy rate and abortion rate between two groups (Table 2).

**Table 2:** Pregnancy outcomes of patients in two groups

	Endometrial injury group (*n* = 100)	Control group (*n* = 100)	*P* value
Biochemical pregnancy rate (*n*, %)	66 (66.00)	49 (49.00)	0.015
Clinical pregnancy rate (*n*, %)	64 (64.00)	48 (48.00)	0.023
Multiple pregnancy rate (*n*, %)	24 (37.50)	9 (18.75)	0.031
Implantation rate (*n*, %)	86/184 (46.74)	56/186 (30.11)	0.001
Live birth rate (*n*, %)	51 (51.00)	36 (36.00)	0.032
Miscarriage rate (*n*, %)	8 (12.50)	5 (10.42)	0.776
Ectopic rate (*n*, %)	1 (1.56)	2 (4.17)	0.575

The endometrial tissues that were obtained by the Pipelle catheter on the third day of the menstrual cycle when introducing the EI showed only superficial layer of the endometrium and proliferative endometrium. The endometrial tissue was confined to the functional zone (Figure S1).

## Discussion

The results of our study suggested that introducing EI during menstruation cycle proceeding frozen–thawed ET cycle has a positive effect on clinical pregnancy and implantation rates for patients who experienced two or more implantation failures.

Successful embryo implantation is a pivotal step in reproduction. This important event can be affected by numerous aspects including several cytokines and growth factors, along with a dialogue between embryo and endometrium.^3^ Endometrial receptivity refers to the condition/capability of endometrium that allows the blastocyst apposition, adhesion and invasion. Endometrial receptivity has been shown to be one of the most important factors that decide the success of implantation. Therefore, many attempts have been tried to improve endometrial receptivity. Among them, there are increasing studies indicate that EI is a very effective method that serves such purpose.^2^, ^3^, ^4^, ^5^, ^6^, ^7^, ^8^, ^9^, ^10^ The procedure of EI is a relatively safe and effective operation, come with low cost and good toleration and usually can be performed in the out‐patient‐setting.

The initial evidence that EI may be helpful for implantation came from in vivo studies.^25^ Later on, numerous studies have proved the effectiveness of EI in improving the outcome in patients undergoing in vitro fertilization.^2^, ^3^, ^4^, ^5^ However, most of the studies that focused on patients with RIF performed EI in the luteal phase of the next proceeding IVF/ICSI cycle. The majority of EI trials have found that EI in luteal phase increased the IVF success rate^.^ 2, 3, 4, 5, 6, 7, 8, 9, 10, 26, 27 The statement above has also been proved by several recent meta‐analysis studies.^10^, ^14^ However, in 2019, another two systematic reviews suggested that current evidence did not fully support performing EI with the purpose of improving success of a first ET attempt.^15^, ^16^ Therefore, the clinical application of EI is still in controversial, especially for the frozen–thawed ET process due to the lack of evidence.

It has been known that EI can remove irregular hyperplasia of endometrial tissue, promote epithelial cells, stromal cells proliferation and differentiation, and enrich the endometrial blood flow.^8^ Up to now, there were six trials focusing on the effect of EI in luteal phase prior to frozen–thawed ET cycle.^17^, ^18^, ^19^, ^28^, ^29^, ^30^ The results of these trials seem contradictive to each other and no firm conclusion can be made whether performing EI in luteal phase of the subsequent frozen–thawed ET cycle is beneficial. Among the studies, only one study focused on the effect of EI in luteal phase before a frozen–thawed ET cycle on live birth rate. Our randomized control study suggested that performing EI during menstruation cycle undergoing frozen–thawed ET cycle had a beneficial effect on our patients.

It is important to point out that the key element that differentiates our study from previous studies is the time of performing EI. Comparing to the conventional method which introduce EI at luteal phase, we performed EI on the third day of the menstrual cycle which shortened the whole process significantly and ensured a more consistent injury due to a thinner endometrial. Another difference was all patients in EI group were subjected to endometrium pathological examination in order to check if the endometrial have corresponded to the early follicular phase and the injury confined to the functional zone. The third difference was that all transferred blastocysts were screened for chromosome abnormality before the transfer. The above differences ensured us obtaining a more consistent cohort compared to previous studies.

There is no consensus regarding the optimal time to perform EI. Most studies performed EI in the luteal phase, proliferative phase or oocyte retrieval. But Karimzade *et al*. showed that performing EI in oocyte retrieval day could have a hazardous effect on the result of ART.^11^ Liu *et al*. compared performing EI in the luteal phase and in the proliferative and suggested that there was no significant difference in terms of clinical outcomes^.^ 31 We believe performing EI in the proliferative phase may influence the characteristic and function of the proliferative endometrium. On the other hand, performing EI on the third day of the menstrual cycle may promote epithelial cells and stromal cells proliferation and differentiation. Our study suggested that EI performed during menstruation cycle undergoing frozen–thawed ET cycle improved the endometrium morphology, the pattern A/B of endometrium at progesterone initiation day was 90% in EI group.

Currently, there are three main hypotheses explain the mechanism of how EI improve the endometrium receptivity: (i) EI might be able to induce endometrial decidualization and pinopode to increase implantation potential.^2^ (ii) The inflammatory reaction induced by EI can increase the secretion of beneficial cytokines and growth factors and eventually upregulated important genes that lead to enhanced endometrial receptivity.^3^, ^32^, ^33^ (iii) EI initiates a wound repair process that creates a lag and serves to better synchronicity of uterus and embryo.^25^, ^34^, ^35^ However, further studies that targeted the mechanisms are needed to better understand this critical question.

In is important to point out that in 2019, Lensen *et al*. conducted a pragmatic, multicenter, open‐label, randomized, controlled trail including 1364 patients, which showed that performing EI between day 3 of the cycle preceding the IVF cycle and day 3 of the IVF cycle did not result in a higher rate of live birth compared to control group for patients treated with IVF.^36^ The incompatibility between their results and our study probably originates from aspects such as differences in population selection (such as ethics, ages, medical history), study design and duration of ET after EI. More studies are needed to further test such ideas.

As far as we are aware, our study is the first to investigate the impact of performing EI during menstruation cycle for patients undergoing frozen–thawed ET after two or more implantation failures. However, due to the limited sample size, some questions such as whether performing EI can reduce the abortion rate were left unanswered. Another possible criticism of our study is that uterine cavity operation on the third day of the menstrual cycle might increase the risk of surgical complications. In order to avoid complications, the operation was kept aseptic. In our study, no patient in the experimental group experienced significant vaginal bleeding, no fever, abdominal pain and other complications were reported. Another important limitation is that both our physicians and patients were not blinded to randomization and the placebo effect is worth considering. Even though our study suggested that performing EI during menstruation cycle is beneficial to the patients, further studies that including performing EI on different menstrual stages are needed to identify the best timepoint of introducing EI.

In summary, our study suggested introducing EI during menstruation cycle proceeding frozen–thawed ET cycle has a positive effect on clinical pregnancy and implantation rates for patients who experienced two or more implantation failures. Our study indicates that introducing EI in menstrual phase undergoing frozen–thawed ET cycle is a feasible, safe and effective method that can improve the clinic outcome. Further studies are necessary to investigate the related mechanisms. Large blinded prospective multicenter studies are also needed to confirm these findings.

## Disclosure

None declared.

## Supporting information


**Figure S1** Supporting information
