# Supplementary figures and images for: Effect of endometrial injury during menstruation on clinical outcomes in frozen–thawed embryo transfer cycles: A randomized control trial
Source: J Obstet Gynaecol Res. 2020 Jan 30;46(3):451–8. doi: 10.1111/jog.14193 (PMC7064918; doi:10.1111/jog.14193)

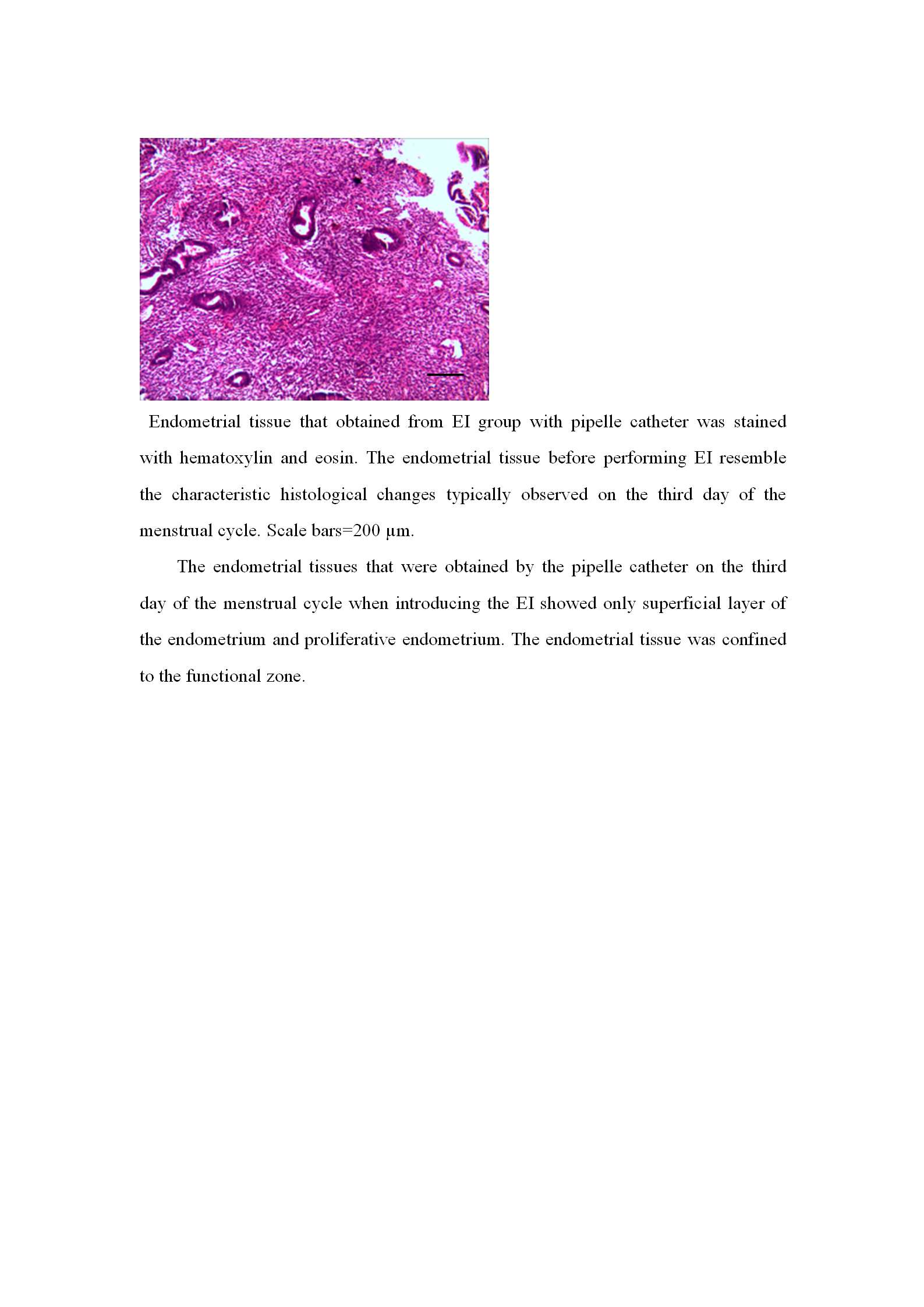

Supplement: Supplementary file 1 — Figure S1 Supporting information [file JOG-46-451-s001.jpg]
